# Predictors and a novel predictive model for intravascular immunoglobulin resistance in Kawasaki disease

**DOI:** 10.1186/s13052-023-01531-7

**Published:** 2023-09-25

**Authors:** Junjie Wang, Xiaohui Huang, Donghao Guo

**Affiliations:** 1 Department of Pediatric Endocrinology, Quanzhou Women’s and Children’s Hospital, Quanzhou, China; 2 Department of Operating Room, Quanzhou Women’s and Children’s Hospital, Quanzhou, China; 3 Department of Cardiology, Shanghai Renji Hospital, School of Medicine, Shanghai Jiaotong University, Shanghai, China

**Keywords:** Kawasaki disease, Intravenous immunoglobulin resistance, Prediction model, Nomogram

## Abstract

**Background:**

Early identification of intravenous immunoglobulin (IVIG) resistance contributes to better management of Kawasaki disease (KD). This study aims to establish an effective prediction model for IVIG resistance in the Chinese population.

**Methods:**

A total of 658 eligible patients diagnosed with KD were enrolled in this study, with 461 in the training cohort and 197 in the validation cohort. The demographic characteristics and potential risk factors were compared between IVIG-responsive and resistant groups. Predictors were selected by the Akaike information criterion. The nomogram’s performance was evaluated by calibration curve, decision curve analysis, and operating characteristic curve.

**Results:**

White blood cell counts (WBC), neutrophil-lymphocyte ratio (N/L ratio), hematocrit (HCT), albumin (ALB), total bilirubin (TBIL), lactate dehydrogenase (LDH), and creatinine (Cr) were detected as predictors of IVIG resistance. A predictive nomogram incorporating these predictors was constructed using the training cohort. The calibration curve and decision curve analysis showed good discrimination and calibration of the proposed nomogram in both training and validation sets, and the area under the receiver operating characteristic curve (AUROC) in both sets was 75.8% and 74.2%, respectively.

**Conclusion:**

This study identified WBC, N/L ratio, HCT, ALB, TBIL, LDH, and Cr as predictors for IVIG resistance in patients with KD. The proposed novel nomogram with a high level of accuracy and reliability may benefit clinical decision-making upon treatment initiation.

## Introduction

Kawasaki disease (KD) is an acute febrile illness of an unknown cause characterized by fever, polymorphic rash, conjunctival congestion, redness of the lip mucosa, swollen neck lymph nodes, erythema, and edema of the extremities. It predominantly affects young children worldwide, with the highest incidence in East Asia. KD has become a leading cause of acquired cardiac disease in children in developed countries 1. Treatment for KD includes intravenous immunoglobulin (IVIG) and high doses of aspirin, which significantly reduce the risk of the coronary artery lesion (CAL) complication (CAL is assessed using echocardiographic z-score. A coronary artery with z-score more than 2 was considered as CAL.). Nonetheless, up to 20% of patients with KD fail to respond to IVIG treatment and remain or relapse fever after 24–48 h following completion of the first dose of IVIG, which is termed IVIG-resistance 2–4. According to the latest American College of Rheumatology (ACR) guideline for the management of KD 1, co-treatment with glucocorticoids or other immunosuppressants is recommended as the initial therapy for children at high risk of IVIG-resistant KD. Hence, identifying children with a high risk of IVIG-resistant KD is warranted for better management of KD.

Although several prediction models for IVIG resistance have been established, the performance of these predictive models lacks consistency in clinical practice in different regions or populations and is limited by the categorization of continuous variables 5, 6. In addition, potential predictors that have been considered vary in different relevant research 7–9. Up to now, there were no effective prediction models for the actual clinical practice in the Chinese population. This cohort study encompassed a comprehensive range of risk factors to evaluate IVIG resistance in children with KD in southeast China, and an effective predictive nomogram, which is a continuous scoring system estimating the risk probability of IVIG resistance for an individual child patient, was established.

## Methods

### Subjects

The study population was patients diagnosed with KD in Quanzhou Children’s Hospital between November 3, 2018 and February 24, 2023. This study was approved by the Ethics Committee of Quanzhou Children’s Hospital. Data were retrieved from the electronic medical records, and all patients were de-identified. The written informed consent was waived because of the retrospective and anonymous nature of the data.

Patients meeting the diagnostic criteria for complete or incomplete KD in accordance with the AHA guidance published in 2017^2^ were included in this study. Patients who did not receive IVIG treatment in Quanzhou Children’s Hospital or those who received glucocorticoids or other immunosuppressants during or prior to the initial IVIG treatment were excluded. The patients were classified into IVIG responsive or resistant groups according to whether patients remained or relapsed fever after 36 h following completion of the first dose of IVIG. The IVIG dosage was 2 g per kilogram of body weight in a single administration. The upper limit for the dosage was capped at 30 g.

### Statistical analysis and nomogram development

The data of demographic features (age and sex) and potential predictors (white blood cell counts, neutrophil-lymphocyte ratio, hematocrit, platelets, sodium ion, albumin, total bilirubin, alanine transaminase, aspartate aminotransferase, gamma-glutamyl transferase, lactate dehydrogenase, creatine kinase-myocardial band, blood urea nitrogen, creatinine, c-reactive protein, procalcitonin, erythrocyte sedimentation rate, DNA damage response, troponin, B-type natriuretic peptide, fever day before treatment, and coronary artery lesion status) were selected for further analysis.

To create a training and validation set, patients were divided based on the time of hospital admission. The earlier 70% of patients were selected as the training set, whereas the remaining 30% from a later period were designated as the validation set.

Missing data were handled by the multiple imputation method using MICE R-Package. Variables with more than 20% missing values were excluded from this study, which are BNP and troponin. Mean values with standard deviations were reported for continuous variables, while frequencies (expressed as percentages) were used for categorical variables.

Patient characteristics were compared between two groups: IVIG-responding and IVIG-resistant, in both the training and validation sets. For continuous baseline characteristics, comparisons were made using the independent Student’s t-test, whereas categorical data were compared using the Chi-square test.

A stepwise backward multivariable logistic analysis was employed to validate these potential risk factors for IVIG resistance using the entire dataset. The stepwise process was evaluated by the Akaike information criterion (AIC) statistics 10. The model with the lowest AIC was selected. Factors such as white blood cell counts, neutrophil-lymphocyte ratio, hematocrit, albumin, total bilirubin, lactate dehydrogenase, and creatinine were included to establish the nomogram model using the training dataset. Subsequently, the predictive model was validated internally using the bootstrap sampling method and externally in the validation dataset.

The sensitivity and specificity of this predictive nomogram and independent predictors were evaluated by operating characteristic curve (ROC). The calibration curve was used to assess the agreement between the predicted result and the observed value. Decision curve analysis was performed to evaluate the clinical utility of this predictive model. Software R (version 4.20, www.r-project.org) was used for the statistical analysis.

## Result

### Baseline characteristics of the patients

A total of 658 eligible patients diagnosed with KD were enrolled in this study. The training and validation datasets included 461 and 197 patients, respectively. Table 1 shows the baseline characteristics upon hospitalization admission of all patients. Participants in the training cohort had more WBC (16.5 ± 6.55 × 10^9/L vs. 15.2 ± 5.09 × 10^9/L, *P* = 0.006) and PCT (368 ± 133 × 10^9/L vs. 328 ± 115 × 10^9/L, *P* < 0.001), lower levels of LDH (320 ± 112 U/L vs. 343 ± 138 U/L, *P* = 0.035) and CK-MB (17.6 ± 14.7 U/L vs. 20.2 ± 15.2 U/L, *P* = 0.048), higher levels of CRP (86.6 ± 56.7 mg/L vs. 68.0 ± 46.5 mg/L, *P* < 0.001) and ESR (68.2 ± 26.8 vs. 58.7 ± 28.9 mg/L, *P* < 0.001), longer fever day before treatment (6.52 ± 2.17 d vs. 6.15 ± 1.87 d, *P* = 0.027), and more proportion with coronary artery lesion (16.9% vs. 8.6%, *P* = 0.008) than those in the validation cohort.

**Table 1:** Demographic and clinical characteristics of the training and validation datasets

Variable	Training (N = 461)	Validation (N = 197)	P value
Month_old			
Mean (SD)	25.9 (20.4)	29.1 (23.8)	0.100
Sex			
Female	169 (36.7%)	74 (37.6%)	0.895
Male	292 (63.3%)	123 (62.4%)	
WBC (×10^9)			
Mean (SD)	16.5 (6.55)	15.2 (5.09)	0.006
N/L ratio			
Mean (SD)	4.04 (3.68)	3.97 (3.75)	0.845
HCT (%)			
Mean (SD)	34.3 (3.40)	34.3 (3.27)	0.825
PLT (×10^9)			
Mean (SD)	368 (133)	328 (115)	< 0.001
Na+ (mmol/L)			
Mean (SD)	136 (2.74)	136 (3.01)	0.820
ALB (g/L)			
Mean (SD)	36.7 (4.21)	37.2 (4.74)	0.202
TBIL (μmol/L)			
Mean (SD)	12.0 (12.5)	12.7 (23.0)	0.696
AST/ALT ratio			
Mean (SD)	1.88 (3.47)	1.66 (1.26)	0.245
GGT (IU/L)			
Mean (SD)	74.7 (85.9)	78.0 (88.2)	0.653
LDH (U/L)			
Mean (SD)	320 (112)	343 (138)	0.035
CK-MB (U/L)			
Mean (SD)	17.6 (14.7)	20.2 (15.2)	0.048
BUN (mmol/L)			
Mean (SD)	3.08 (2.72)	3.13 (1.16)	0.736
Cr (μmol/L)			
Mean (SD)	20.8 (11.4)	21.0 (9.05)	0.797
CRP (mg/L)			
Mean (SD)	86.6 (56.7)	68.0 (46.5)	<;0.001
PCT (ng/ml)			
Mean (SD)	2.87 (9.34)	4.47 (16.1)	0.194
ESR (mm/h)			
Mean (SD)	68.2 (26.8)	58.7 (28.9)	< 0.001
DDR (mg/L)			
Mean (SD)	1.55 (2.13)	1.62 (2.16)	0.714
fever_day_before_treatment			
Mean (SD)	6.52 (2.17)	6.15 (1.87)	0.027
Coronary artery lesion			
No	383 (83.1%)	180 (91.4%)	0.008
Yes	78 (16.9%)	17 (8.6%)	

### Comparison of IVIG responsive and resistant patients

Table 2 shows the univariable logistic analysis results for IVIG resistance. The differences in WBC, N/L ratio, PLT, Na+, ALB, TBIL, GGT, BUN, CRP, PCT, and DDR were significant in the training cohort, whereas the levels of ALB and Cr differed significantly in the validation cohort. All potential risk factors were evaluated by a stepwise backward multivariable logistic analysis. WBC, N/L ratio, HCT, ALB, TBIL, LDH, and Cr were detected by the Akaike information criterion (AIC) as potential predictors for IVIG resistance, which were used for subsequent nomogram development.

**Table 2:** Comparison of demographic and clinical characteristics between patients with IVIG resistant and non-resistant KD.

Variable	Training set (N = 461)			Test set (N = 197)		
	IVIG-responsive (N = 417)	IVIG-resistant (N = 44)	P-value	IVIG-responsive (N = 161)	IVIG-resistant (N = 36)	P-value
Month_old						
Mean (SD)	25.5 (19.3)	30.3 (28.6)	0.278	27.8 (22.2)	35.1 (29.3)	0.167
Sex						
Female	154 (36.9%)	15 (34.1%)	0.836	60 (37.3%)	14 (38.9%)	1.000
Male	263 (63.1%)	29 (65.9%)		101 (62.7%)	22 (61.1%)	
WBC (×10^9)						
Mean (SD)	16.7 (6.65)	14.6 (5.19)	0.013	15.5 (5.11)	14.0 (4.94)	0.122
N/L ratio						
Mean (SD)	3.77 (3.27)	6.50 (5.89)	0.004	3.62 (2.89)	5.55 (6.10)	0.071
HCT (%)						
Mean (SD)	34.4 (3.37)	34.0 (3.73)	0.573	34.5 (2.99)	33.3 (4.20)	0.106
PLT (×10^9)						
Mean (SD)	373 (132)	322 (135)	0.022	331 (109)	317 (141)	0.571
Na+ (mmol/L)						
Mean (SD)	136 (2.68)	135 (3.01)	0.003	136 (2.87)	136 (3.61)	0.853
ALB (g/L)						
Mean (SD)	36.8 (4.18)	35.3 (4.30)	0.026	37.5 (4.72)	35.7 (4.61)	0.041
TBIL (μmol/L)						
Mean (SD)	11.1 (10.9)	20.8 (20.7)	0.004	10.3 (9.96)	23.5 (48.5)	0.111
AST/ALT ratio						
Mean (SD)	1.93 (3.61)	1.38 (1.43)	0.051	1.64 (1.22)	1.74 (1.44)	0.724
GGT (IU/L)						
Mean (SD)	69.9 (79.8)	120 (124)	0.011	74.3 (84.6)	94.8 (102)	0.269
LDH (U/L)						
Mean (SD)	317 (113)	345 (102)	0.093	336 (134)	381 (158)	0.121
CK-MB (U/L)						
Mean (SD)	17.6 (15.2)	17.6 (9.70)	0.965	19.8 (14.5)	22.0 (17.9)	0.496
BUN (mmol/L)						
Mean (SD)	2.99 (2.71)	3.87 (2.63)	0.041	3.10 (1.19)	3.25 (1.01)	0.430
Cr (μmol/L)						
Mean (SD)	20.3 (9.41)	25.3 (22.5)	0.156	20.1 (7.40)	25.2 (13.7)	0.038
CRP (mg/L)						
Mean (SD)	84.5 (55.8)	107 (61.1)	0.025	65.2 (46.6)	80.8 (44.8)	0.067
PCT (ng/ml)						
Mean (SD)	2.37 (9.00)	7.67 (11.1)	0.004	4.19 (16.2)	5.73 (15.8)	0.602
ESR (mm/h)						
Mean (SD)	68.3 (27.0)	66.8 (24.7)	0.708	58.7 (27.7)	58.9 (34.3)	0.970
DDR (mg/L)						
Mean (SD)	1.47 (2.08)	2.27 (2.50)	0.047	1.48 (2.14)	2.21 (2.17)	0.075
fever_day_before_treatment						
Mean (SD)	6.57 (2.12)	6.00 (2.51)	0.15	6.16 (1.66)	6.08 (2.66)	0.866
Coronary artery lesion						
No	349 (83.7%)	34 (77.3%)	0.385	148 (91.9%)	32 (88.9%)	0.796
Yes	68 (16.3%)	10 (22.7%)		13 (8.1%)	4 (11.1%)	

### A predictive nomogram for IVIG resistance

**Fig. 1:**
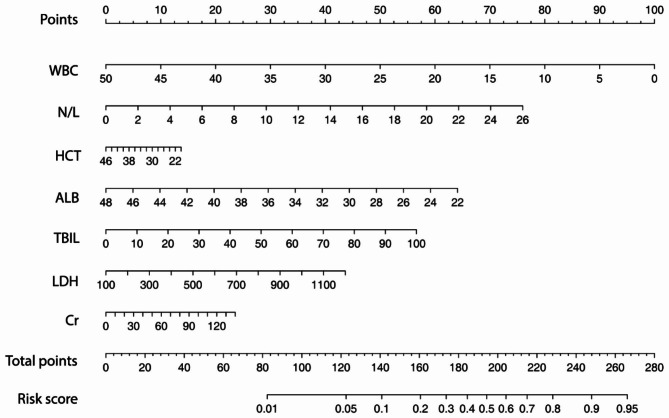
The predictive nomogram for IVIG resistance in KD patients

The potential independent risk factors for IVIG resistance were included to establish the predictive nomogram using the training dataset as shown in Fig. 1. Greater cumulative points, calculated by summing the assigned scores for each predictor in the nomogram, were associated with an increased risk of IVIG resistance.

**Fig. 2:**
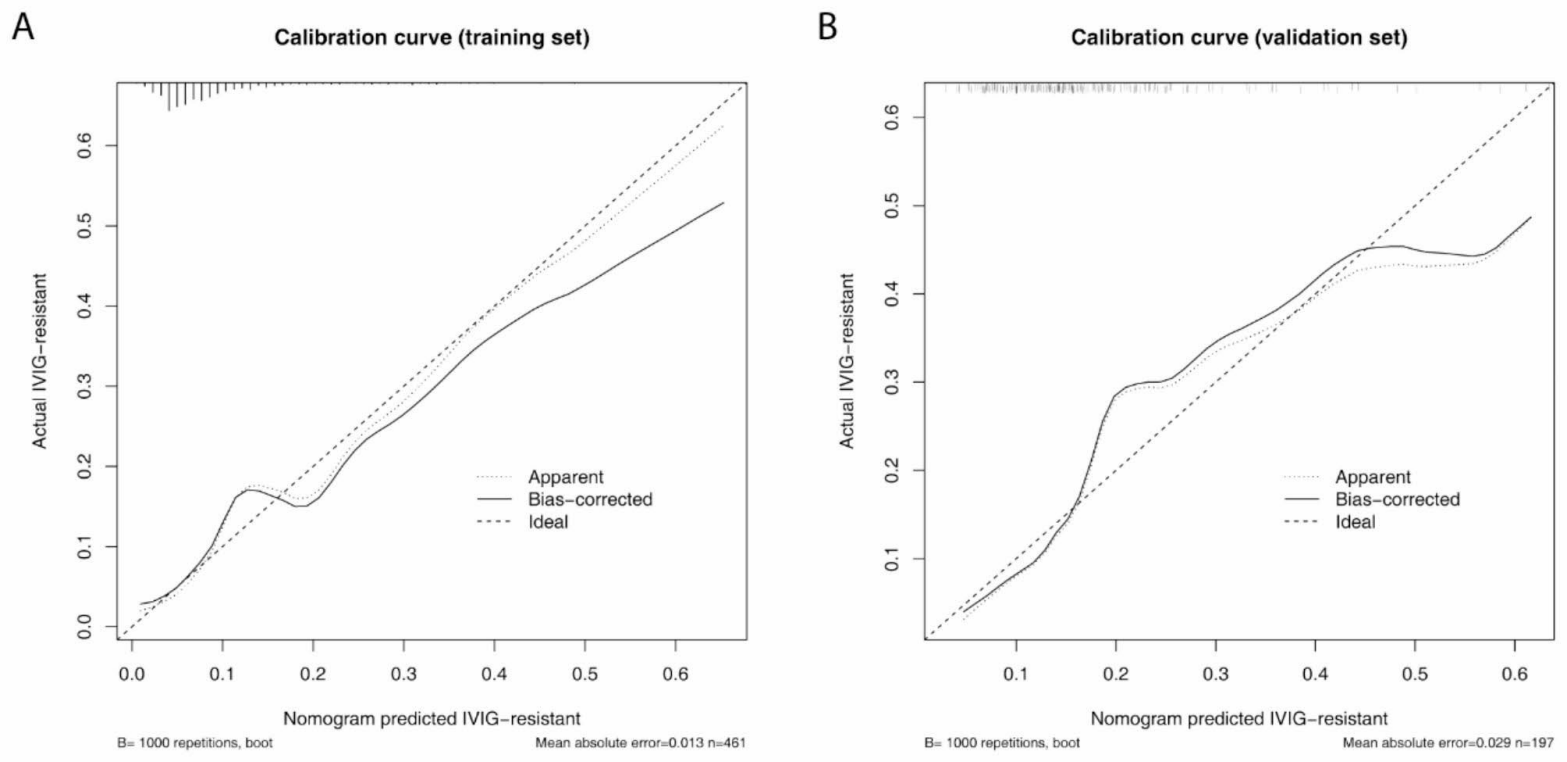
Calibration curve analysis of the predictive nomogram model in the (**A**) training and (**B**) validation datasets

Calibration curves were plotted to evaluate the predictive accuracy of the nomogram. Figure 2 A and B show the consistency of the prediction and actual observation in both training and validation cohorts, indicating the good calibration ability of this predictive nomogram.

### Clinical use of the nomogram

**Fig. 3:**
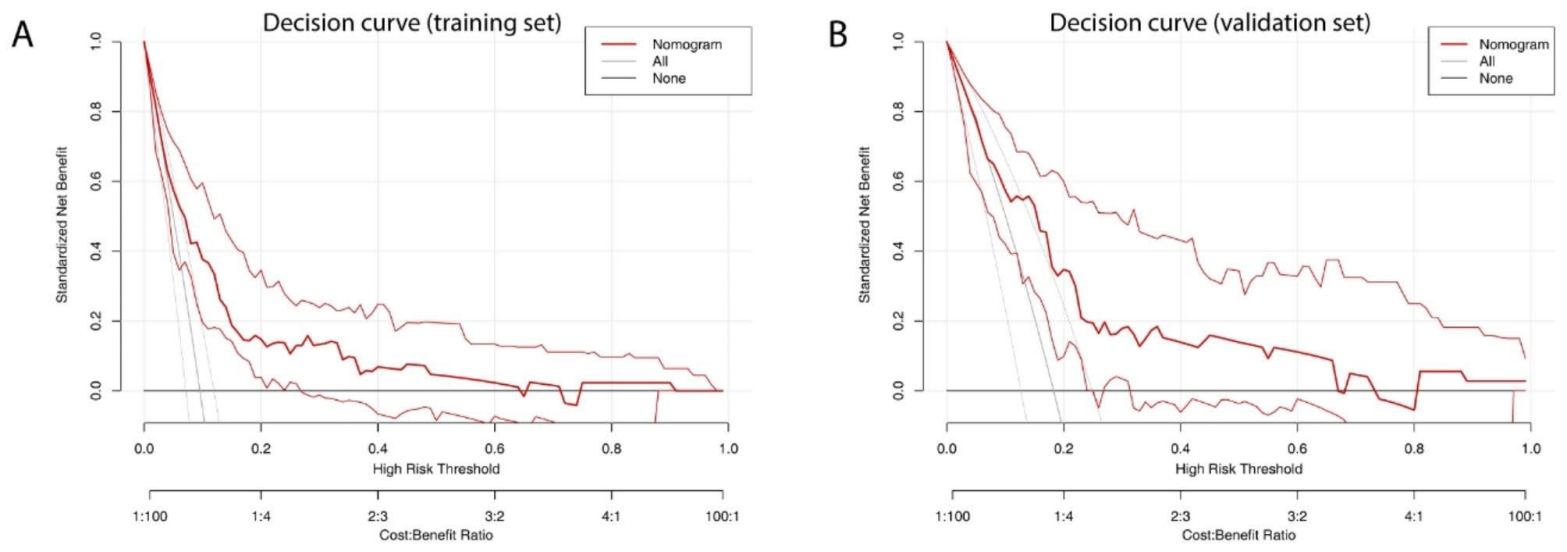
Decision curve analysis of the predictive model in the training and validation dataset

Regarding the clinical use of this nomogram, decision curve analysis was conducted to estimate the net benefit of this model by comparing the difference between the number of true and false positive results. As shown in Fig. 3A and B, when the threshold probabilities ranged between 0.1 and 0.6, medical intervention guided by the nomogram can gain more net benefit than the “treat all” and “treat none” strategies, suggesting the clinical usefulness of the predictive nomogram for IVIG resistance.

### Sensitivity and specificity analysis

**Fig. 4:**
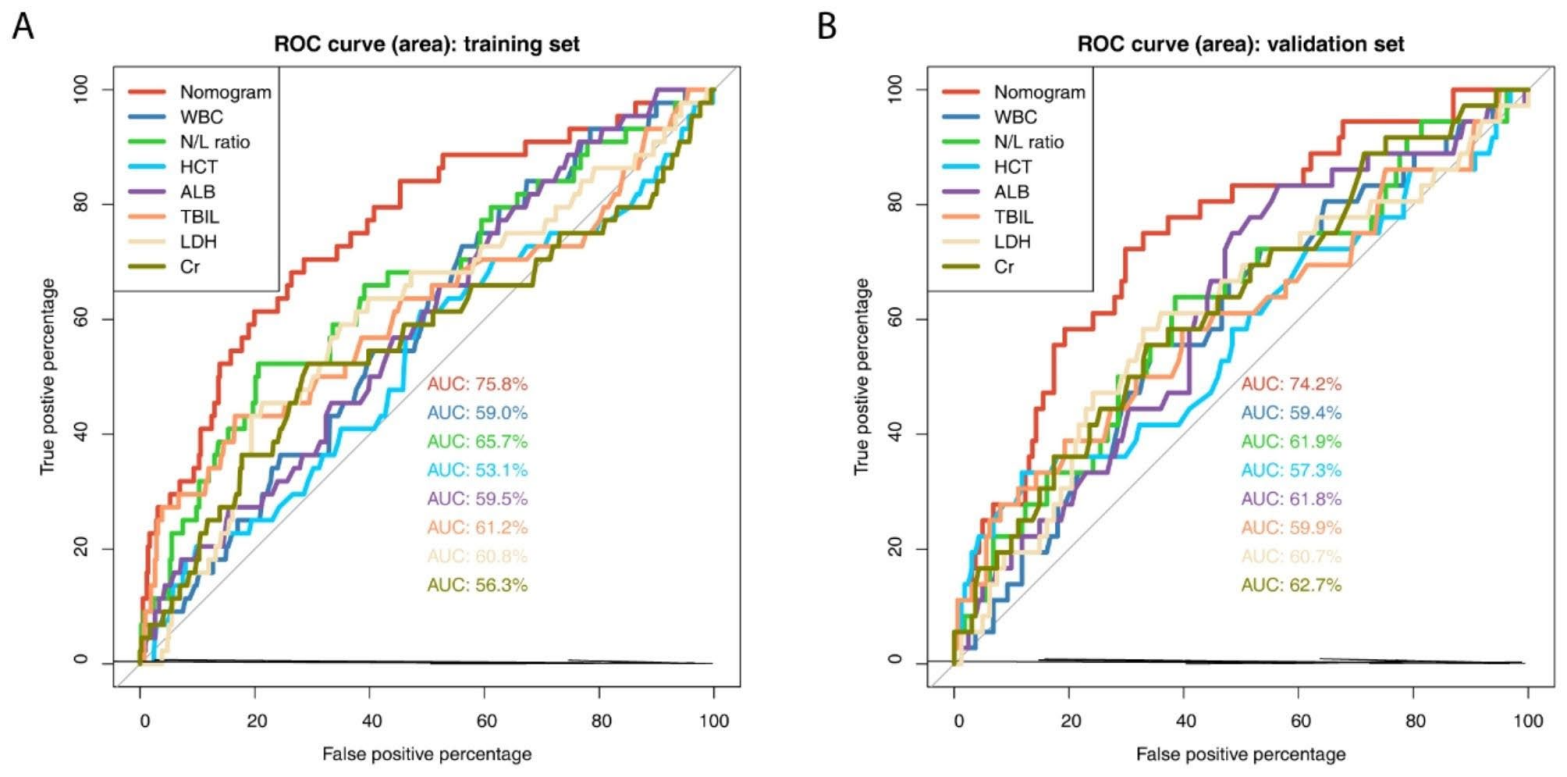
ROC curves of the predictive nomogram and the risk predictors in the training and validation cohorts

The sensitivity and specificity of the nomogram and each predictor were assessed respectively using the receiver operating characteristic curve (ROC curve). As shown in Fig. 4A, the area under the receiver operating characteristic curve (AUROC) of the nomogram was 75.8%, which was greater than those of WBC (59%), N/L ratio (65.7%), HCT (53.1%), ALB (59.5%), TBIL (61.2%), LDH (60.8%), and Cr (56.3%) in the training dataset. Consistently, as shown in Fig. 4B, the AUROC of the nomogram was 74.2%, which was greater than those of each predictor (WBC 59.4%, N/L ratio 61.9%, HCT 57.3%, ALB 61.8%, TBIL 59.9%, LDH 60.7%, and Cr 62.7%). The AUROC results from both training and validation sets showed that the predictive nomogram had better discrimination and calibration than each individual predictor, implying its good prediction performance.

## Discussion

Combinations of initial IVIG therapy and glucocorticoids or other immunosuppressants have been reported to effectively reduce the incidence of the coronary artery lesion (CAL) complication for children at high risk of IVIG-resistant KD 11–13, and therefore are recommended by the latest American College of Rheumatology (ACR) guideline for management of KD 1. Hence, it is imperative to develop an accurate predictive model for IVIG resistance. However, the inconsistency of the scoring systems in clinical practice in different regions or populations limits its application in general population. For example, the Japanese scoring system, Kobayashi, Egami and Sano, have a high predictive value in Japanese population but exhibit low sensitivities or specificities in other populations 7, 8, 14. It is of great significance in developing an effective scoring system for IVIG resistance in the Chinese population. Recently, researchers have developed several prediction models based on the Chinese population 15, 16. Nonetheless, these models have limitations, and none of them were officially accepted by any formal medical association. The continuous variables in these models were converted to categorical variables, losing within-category information so as to reduce the prediction accuracy. This study, in contrast, kept the continuous variables continuous. As the continuous variables included in this nomogram, such as WBC, N/L ratio, HCT, ALB, TBIL, LDH, and Cr, have been reported to have a monotonicity relation with IVIG resistance 17–19, this makes our predictive model more precise and reliable 20.

The nomogram is an invaluable computational model for prognostic prediction due to its ability to provide a visual representation of a statistical predictive model and generate a precise numerical probability for clinical events 21. It surpasses the conventional method that relies on odds ratios, offering enhanced accuracy 22. Herein, we developed a novel nomogram capable of estimating the risk probability of IVIG resistance in patients with KD. The parameters utilized in our model are readily accessible in medical centers, enabling a quick assessment of patients with KD before the determination of initial treatment strategies.

This study identified WBC, N/L ratio, HCT, ALB, TBIL, LDH, and Cr as significant predictors for IVIG resistance. WBC, as an essential serum biomarker related to inflammation and infection, has previously been reported to be associated with IVIG resistance in patients with KD. However, it is debatable how these parameters were correlated to IVIG resistance, and some studies even showed opposite results 23–25. In this study, the WBC was negatively correlated to the IVIG resistance risk. Regarding the debatable results from different studies, we assume that there might be some confounding variables that affect both WBC and IVIG resistance risk, which requires further investigation. On the other hand, the significance of N/L ratio, HCT, ALB, TBIL, LDH, and Cr as predictors of IVIG resistance was further confirmed in this study, whereas other characteristics, which were previously reported to be risk factors, were not found to be significantly associated with IVIG resistance, probably attributing to the sample size, different populations, or different local clinical practices.

Notably, recent studies reported that incomplete KD was closely related to IVIG responsiveness 16, 26. Although incomplete KD was not included as an independent risk factor in this study, the factors in the diagnostic criteria of complete or incomplete KD were analyzed. Nonetheless, further investigation on the association between incomplete KD and IVIG resistance is required.

Meanwhile, there are several limitations of this study. First, the present study is a retrospective observational study rather than a randomized controlled clinical study, which does not possess strong capabilities to establish causality 27. Second, other confounding variables may be present that were not measured. Third, this study, as a single-center study, is more susceptible to local clinical practices, limiting the validity and generalizability of our findings.

## Conclusions

Overall, this study identified WBC, N/L ratio, HCT, ALB, TBIL, LDH, and Cr as the independent risk factors for IVIG resistance in patients with KD. The proposed novel nomogram with a high level of accuracy may benefit the clinical decision-making upon treatment initiation.

## Data Availability

The datasets used and analyzed in the current study are available from the corresponding author upon reasonable request.

## List of abbreviations

ACR: American College of Rheumatology
AIC: Akaike information criterion
ALB: Albumin
ALT: Alanine transaminase
AST: Aspartate aminotransferase
AUROC: Receiver operating characteristic curve
BNP: B-type natriuretic peptide
BUN: Blood urea nitrogen
CAL: Coronary artery lesion
CK-MB: Creatine kinase-myocardial band
Cr: Creatinine
CRP: C-reactive protein
DDR: DNA damage response
ESR: Erythrocyte sedimentation rate
GGT: Gamma-glutamyl transferase
HCT: Hematocrit
IVIG: Intravenous immunoglobulin
KD: Kawasaki disease
LDH: Lactate dehydrogenase
N/L ratio: Neutrophil-lymphocyte ratio
Na+: Sodium ion
PCT: Procalcitonin
PLT: Platelets
ROC: Operating characteristic curve
TBIL: Total bilirubin
WBC: White blood cell count
